# Characteristics of Near-Death Experiences Memories as Compared to Real and Imagined Events Memories

**DOI:** 10.1371/journal.pone.0057620

**Published:** 2013-03-27

**Authors:** Marie Thonnard, Vanessa Charland-Verville, Serge Brédart, Hedwige Dehon, Didier Ledoux, Steven Laureys, Audrey Vanhaudenhuyse

**Affiliations:** 1 Coma Science Group, Cyclotron Research Centre and Neurology Department, University and University Hospital of Liège, Liège, Belgium; 2 Cognitive and Behavioral Neurosciences Centre, University of Liège, Liège, Belgium; Cuban Neuroscience Center, Cuba

## Abstract

Since the dawn of time, Near-Death Experiences (NDEs) have intrigued and, nowadays, are still not fully explained. Since reports of NDEs are proposed to be imagined events, and since memories of imagined events have, on average, fewer phenomenological characteristics than real events memories, we here compared phenomenological characteristics of NDEs reports with memories of imagined and real events. We included three groups of coma survivors (8 patients with NDE as defined by the Greyson NDE scale, 6 patients without NDE but with memories of their coma, 7 patients without memories of their coma) and a group of 18 age-matched healthy volunteers. Five types of memories were assessed using Memory Characteristics Questionnaire (MCQ – Johnson et al., 1988): target memories (NDE for NDE memory group, coma memory for coma memory group, and first childhood memory for no memory and control groups), old and recent real event memories and old and recent imagined event memories. Since NDEs are known to have high emotional content, participants were requested to choose the most emotionally salient memories for both real and imagined recent and old event memories. Results showed that, in NDE memories group, NDE memories have more characteristics than memories of imagined and real events (p<0.02). NDE memories contain more self-referential and emotional information and have better clarity than memories of coma (all ps<0.02). The present study showed that NDE memories contained more characteristics than real event memories and coma memories. Thus, this suggests that they cannot be considered as imagined event memories. On the contrary, their physiological origins could lead them to be really perceived although not lived in the reality. Further work is needed to better understand this phenomenon.

## Introduction

After being close to death, some people will report having had an out-of-body experience, having seen a bright light or being passed through a tunnel; all well-known elements of the famous “Near-Death Experience” (NDE). NDEs are defined as “*profound psychological events with transcendental and mystical elements, typically occurring to individuals close to death or in situations of intense physical or emotional danger*” [1]. Moreover, NDEs or NDE-like do not only occur under the circumstances mentioned in this definition. Indeed, other experiences do at least share characteristics of NDEs (e.g. certain kinds of epileptic seizures [2]). In addition, the literature shows that many individuals having had NDEs were not physically in danger of death suggesting that the perception, on its own, of the risk of death seems to be important in eliciting NDEs [3].

Since the dawn of time, NDEs have intrigued by their paranormal appearance and their study has led to a wide variety of biological as well as psychological or transcendental theories. Although some theories can explain some components of NDEs (e.g. out-of-body experiences (OBE), seeing a bright light, life review, and so on; for review see [4], [5]), none of these can explain, single-handedly, the entire phenomenon.

It has been proposed that reports of NDEs could be memories of events that never happened or altered memories of real events [6], [7]. Notably, Blackmore [6] has proposed that NDEs have both physiological and psychological mechanisms. According to her model, the core experience is biologically determined, caused by different awry normal mechanisms, but the interpretation and details can be influenced by the experiencer's prior knowledge and beliefs, particularly in situations of physical or psychological threat. The resulting memories of this experience could thus be, at least in part, imagined. Memories of imagined events have been shown to contain less phenomenological characteristics than memories of real events. Indeed, several studies [8], [9], [10] have shown that memories of imagined events contain less perceptual (i.e., visual, auditory, gustatory and olfactory sensations), temporal and spatial details, and emotional information as assessed by the Memory Characteristics Questionnaire (MCQ [10], a questionnaire built to analyze phenomenological characteristics of real and imagined memories).

The aim of the present study was to determine the phenomenological characteristics of NDEs using the MCQ [10] and compare them with those of memories of real and imagined past events in three groups of coma survivors: patients with NDE as defined by the Greyson NDE scale [11], patients with memories of their coma without NDE, patients without memory of their coma) and an age-matched control group of healthy volunteers.

Participants were ask to recall five types of past events that were assessed using MCQ: recent (1) and old (2) *real* events memories (e.g. memories of particular events, visit, etc.); recent (3) and old (4) *imagined* events memories (e.g. memories of dream or of unfulfilled intention) and target memories (5; i.e., NDE memories for the NDE group, coma memories for the coma memory group and first childhood memories for the no memory group and the healthy controls). Participants were instructed to recall the most emotionally salient memories for both real and imagined recent and old event memories.

It has been suggested that reports of NDE could be reports of imagined events [7]. For some authors, only some components of NDE's are imagined (e.g. [6]). On the contrary, some NDE experiencers report their experience as “real” or even “realer than real” [12]. We here propose to assess NDE memory characteristics as compared to real and imagined event memories.

## Results

### Effects of time and source

In order to verify the properties of the MCQ, as previously described by Johnson et al. [10], we tested the effect of time (recent memories should have more characteristics than old ones) and source (real memories should have more characteristics than imagined ones) for the comparison of real and imagined memories in our sample. We found an effect of time for both real and imagined event memories in the coma memory group (*p* = 0.046 and *p* = 0.027, respectively) and the control group (both *p*<0.001). The NDE memory group and the “no memory” group showed an effect of time for real event memories (*p* = 0.012, *p* = 0.018 respectively) but not for imagined memories (*p* = 0.833, *p* = 0.310, respectively). A source effect was found in all groups for recent and old memories (NDE group: both *p* = 0.012; coma memory group: both *p* = 0.027; no memory group: *p* = 0.018 and *p* = 0.028, respectively; control group: both *p*<0.001).

### Comparison of target memories with real and imagined memories

In NDE memory group, target memories (i.e., NDEs) have higher MCQ total scores than all other memories (*p*<0.05). Target memories in the coma memory group (i.e., memories of the coma period not considered as NDEs according to the Greyson's scale [11]) had lower MCQ total scores than real recent event memories (*p* = 0.028) but were not different from imagined recent or old memories or real old memories. Target memories of the no memory group (i.e., first childhood memories) were different from real old or recent memories (*p*<0.05) but not from imagined old or recent event memories. Finally, target memories of the control group (i.e., first childhood memories) were different from all assessed event memories (*p*<0.05).

### Group effect on memories

**Figure 1:**
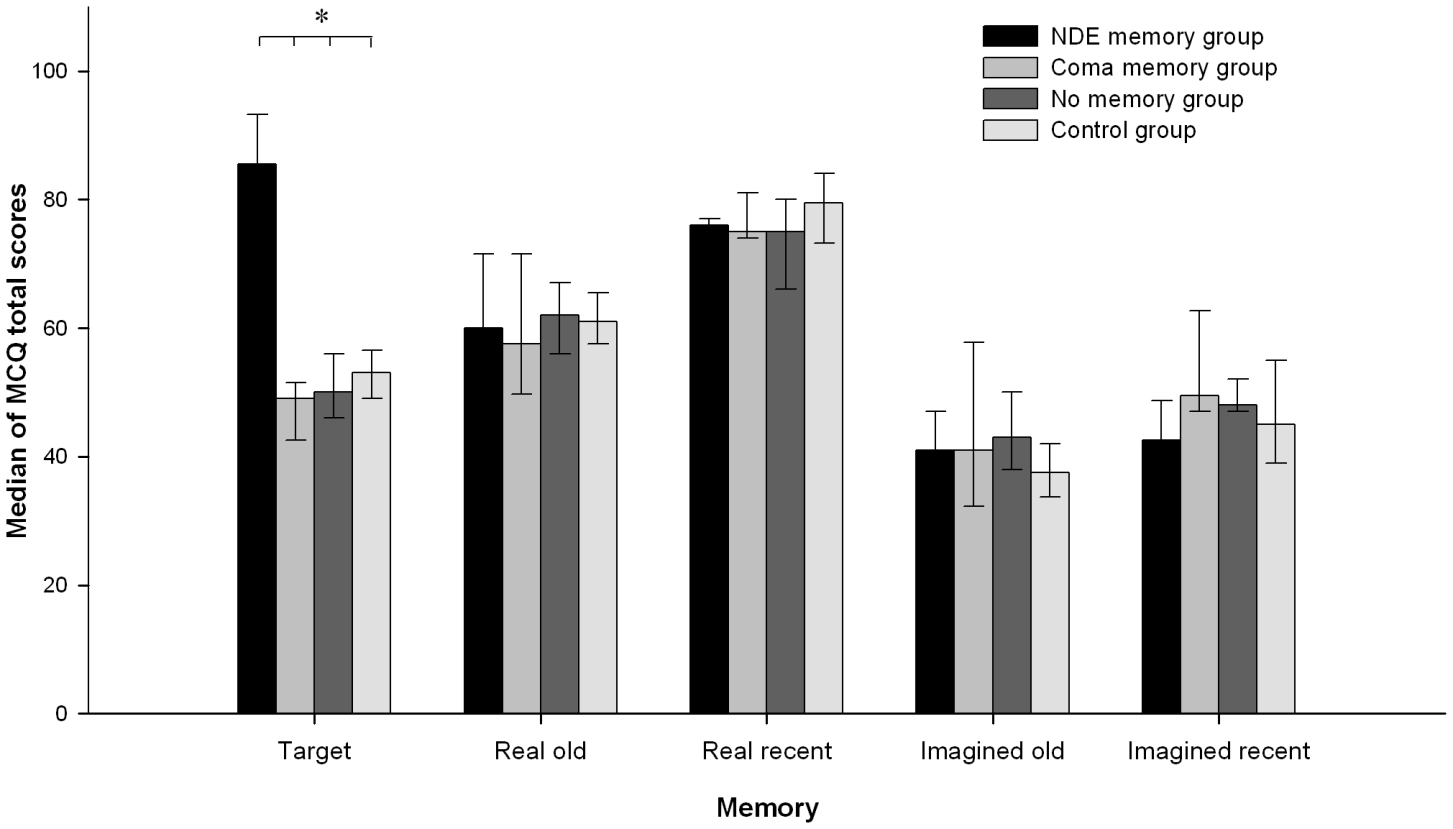
Memory Characteristics Questionnaire (MCQ [10]) total scores for each assessed memory (median and interquartile ranges; *p<0.05). Note that NDEs show significantly more characteristics as compared to the assessed target memories in the coma, “no memory” and control groups.

**Figure 2:**
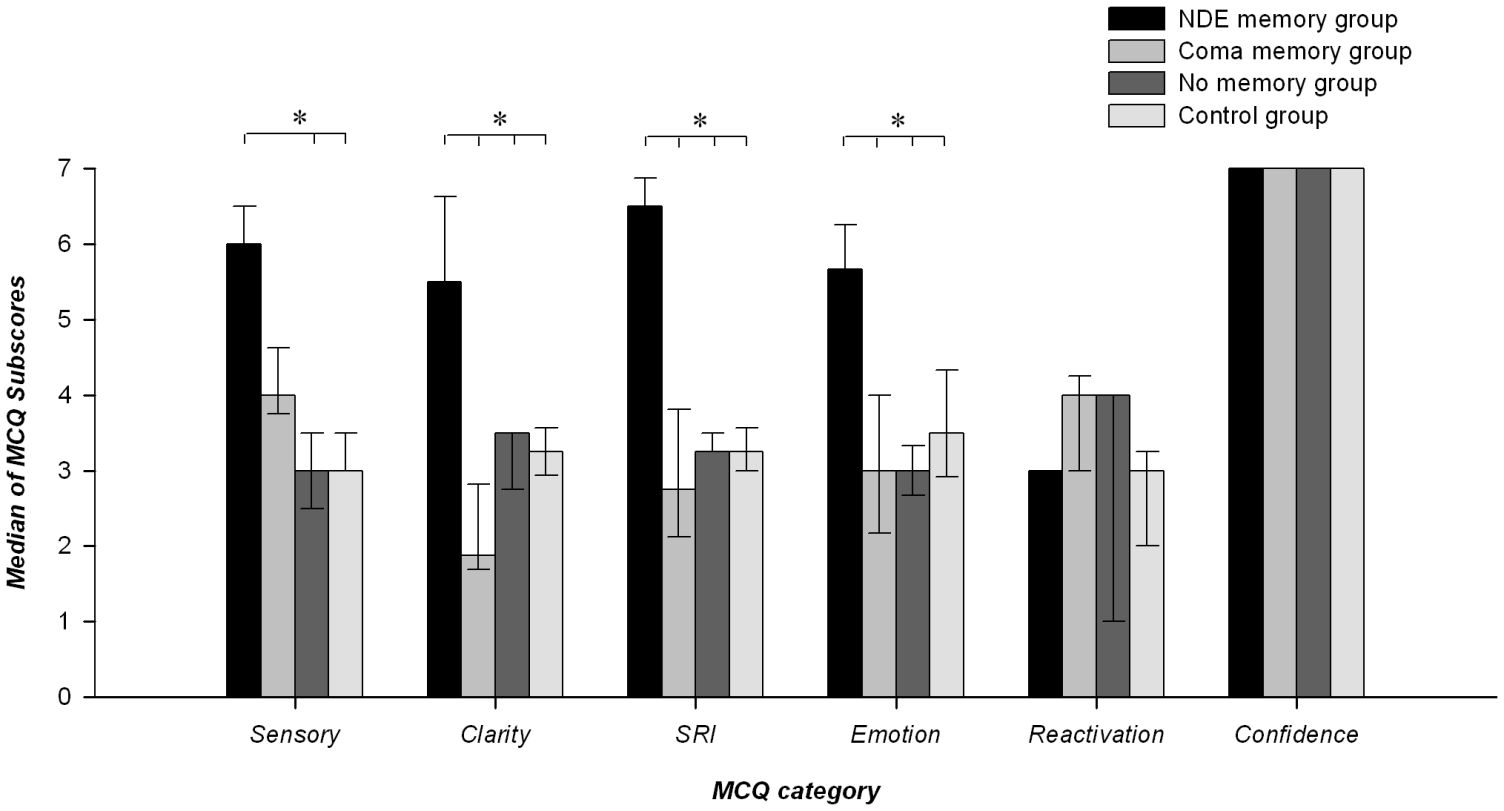
Memory Characteristics Questionnaire (MCQ [10]) subscores (median and interquartile ranges; *p<0.05). Note a significantly higher amount of memory characteristics for sensory, clarity, self-referential information (SRI) and emotion categories for NDEs as compared to target memories in the coma, “no memory” and control groups.

According to the MCQ total scores, a group effect was observed for target memories but not for other assessed memories (Figure 1; *H*(3, N = 39) = 20.57; *p*<0.001). Post-hoc analyses identified higher MCQ total scores only for NDE as compared to other target memories (*p*<0.005). The analysis of category subscores showed a group effect on target memories for sensory details, memory clarity, self-referential and emotional information items (*p*<0.005 – Figure 2) but not for other categories (reactivation frequency and confidence in their own memory). For target memories, NDE and coma memory groups reported more sensory characteristics than no memory and controls groups (all p<0.005). For sensory characteristics, there was no difference between NDE and coma memory groups and between no memory and control groups. Concerning the memory clarity, NDE memories showed more characteristics than target memories of all other groups (*p*<.005); coma memories contained less characteristics than the control group's childhood memories (*p*<.005); no differences were found between target memories of the no memory and control groups. In addition, for the self-referential information category, only NDE memories showed more characteristics than target memories in all other groups (*p*<.005). For emotional information items, only NDE memories showed more characteristics than target memories of all other groups (*p*<.05).

## Discussion

Our phenomenological characterization of NDEs, by means of the MCQ, shows that NDEs cannot be considered as typical imagined event memories. The observation that we identified a higher amount of memory characteristics for recent, as compared to old, and for real as compared to imagined events, are in line with previous work by Johnson et al. [10]. However, in the “NDE” and “no memory” groups, we showed no significant effect of time on the amount of memory characteristics. Given that real and imagined memories were not different between groups, this lack of time-effect could be explained by the low sample size in our study.

Within-group analyses showed that NDE experiencers reported more characteristics for NDEs than for real and imagined memories (both old and recent), contrary to what was observed for target memories in the other groups. It is important to stress that, despite our efforts to ask participants to choose a memory with matched emotionality to the target event, NDEs seem to be of extremely high emotional content, as reflected by MCQ assessment, possibly biasing within-NDE-group comparisons. Between-group analyses revealed that NDE memories showed more characteristics than other target memories (i.e. coma memories, first childhood memories of no memory group and healthy controls). These results highlight the point that NDEs seem unique, unrivalled memories. The finding that NDEs are emotionally strong and closely linked to subjects' own identity is in line previous reports (e.g. [13], [14]). Memories of NDEs contained both more emotional and self-referential information than other target memories. The higher emotional value of NDE memories could explain the greater amount of sensory details in these memories. Indeed, previous studies have shown that emotional value enhances the amount of sensory details in memories [15]. On the other hand, such a strong emotional event is more likely to be repeatedly rehearsed (internally and/or in re-telling to others) and thus could increase the amount of characteristics. However, the results of the present study did not show any effect of reactivation depending on the memory.

Furthermore, the greatest overall amount of characteristics in NDE memories could be explained by the fact that self-referential information can enhance recalling performance. In fact, this kind of information could permit an easier encoding, organization, and enrichment by extended knowledge, leading to a subjective and detailed episodic representation of the original event and associated thoughts and feelings [16]. However, it has to be noted that this effect is generally reported in comparison with memories related to another person reference, unlike here. But if we assume that NDEs could have a stronger importance for personal identity and can be more closely linked to the subject's identity than other experiences (as NDEs have already been reported having short and long term consequences on a patient's life [13]), then it is likely that self-referential content could have an effect on the overall characteristics of NDE memories. Because of its emotionality and consequentiality, NDE memories could meet the definition of “*flashbulb memories*”. Indeed, a highly emotional, personally important, and surprising event can benefit from a preferential encoding that makes them more detailed and longer-lasting than everyday memories and that leads to what is called a *flashbulb memory* [17].

Interestingly, NDE memories in this study contained more characteristics than coma memories, suggesting that what makes the NDEs “unique” is not being “near-death” but rather the perception of the experience itself. Indeed, even if being “near-death” often is traumatizing experience, this does not necessarily explain why NDE and coma memories are different. This is in line with the hypothesis that the core components of a NDE are neurophysiologically determined [4], [18]. If we assume that some physiological mechanisms can account for NDEs (e.g. OBEs caused by a deficient multisensory integration at the right [19], [20], [21] or left [22] temporo-parietal junction or feeling the presence of another (deceased) person possibly caused by left temporo-parietal junction dysfunction [20]), then the subject really perceived these phenomena, albeit not corresponding to occurring events in reality. At this point, NDEs can meet the definition of hallucinations : “Any percept-like experience which (a) occurs in the absence of an appropriate stimulus, (b) had the full force or impact of the corresponding actual (real) perception, and (c) is not amenable to direct and voluntary control by the experiencer” [23]. Note that hallucinations are recognized to most often have pathophysiological or pharmacological origins, as we hypothesize, also is the case for NDEs. As for hallucinations, NDEs present a real perceptual bias (due to physiological mechanisms taking place during NDEs) and can include as many characteristics as real event memories. In addition, the effects of emotional and self-referential values of the NDE could make it a kind of “super-real” memory containing even more characteristics than real event memories. Considering together the concept of flashbulb memories and the similarity of NDEs with hallucinations, the higher amount of characteristics for NDEs that was here observed suggest that the memories of NDEs are flashbulb memories of hallucinations.

In conclusion, the present study shows that NDE memories have more characteristics than any kind of memory of real or imagined events and of other memories of a period of coma or impaired consciousness following an acquired severe brain dysfunction. In our opinion, the presented data demonstrate that NDEs cannot be considered as imagined events. We rather propose that the physiological origins of NDEs lead them to be really perceived although not lived in reality (i.e., being hallucination- or dream-like events), having as rich characteristics as memories of real events. The amount of characteristics of NDE memories probably is further enhanced by their here-identified high emotional and self-referential values. This suggests that memories of NDEs are flashbulb memories of really perceived hallucinations. Although the similarities of NDEs with hallucinations are striking, further research is needed to characterize the relationship between these phenomena more precisely. Finally, additional neuroimaging studies are needed in order to better understand the neural signature of NDEs.

## Method

### Subjects

We included 21 patients who suffered from an acute brain insult and recovered from a coma (defined as Glasgow Coma Scale total score <8 [24]) (6 traumatic; age 48±14 years) studied 14±99 months after the insult. Patients were divided in 3 groups: (1) reporting memories of a NDE (Greyson NDE score≥7 [11]; N = 8; 46±19 years; 93±87 months after insult; 3 traumatic, 2 anoxic, 1 hemorrhagic and 2 metabolic etiologies), (2) reporting memories associated to the coma and intensive care period but without NDE (Greyson NDE score<7; N = 6; 49±8 years; 147±120 months post-insult; 3 traumatic and 3 anoxic etiologies), (3) reporting no memories of their coma (N = 7; 49±16 years, 140±93 months post-insult; 2 traumatic, 2 anoxic, 2 encephalopathy and 1 hemorrhagic etiologies). Results were compared with 18 healthy control subjects (55±15 years). The study was approved by the Ethics Committee of the Medical School of the University of Liege. Written informed consent was obtained from all the subjects enrolled in the study.

### Material

We assessed the participants' memories characteristics using a modified version (Table 1) of the Memory Characteristics Questionnaire (MCQ) [10]. This version encompasses 15 items assessing 6 categories of memory characteristics: sensory details, memory clarity, self-referential and emotional information, reactivation frequency, and confidence in their own memory. We asked participants to recall several past events that were assessed with MCQ. Five types of memories were assessed: (1) recent and (2) old real events memories (e.g. memories of a social occasion, visit or travel), (3) recent and (4) old imagined events memories (e.g., memories of past dreams, fantasy, or unfulfilled intentions) and (5) target memories (i.e., NDE memories for the NDE group, coma memories for the coma memory group and first childhood memories for the no memory group and healthy controls). First childhood memories were chosen as target control memory for both no memory and healthy control because of its susceptibility to be an imagined/implanted event [25]. Participants were invited to match emotional value and personal importance for real and imagined recent and old memories with target memories. For old memories (both real and imagined) participants were invited to select events with similar temporal precedence as for the NDEs or coma related memories.

**Table 1:** Modified version of Memory Characteristics Questionnaire ([10], adapted from D'argembeau & Van der Linden [26]).

Category	Characteristic	Modified version of Memory Characteristics Questionnaire (adapted from [26])
***Sensory***	*Visual details*	My memory for this event invlolves visual details: 1 = none, 7 = a lot
	*Other sensory details*	My memory for this event involves other sensory details (sounds, smells, and/or tastes): 1 = none, 7 = a lot
***Clarity***	*Feeling of re-experiencing*	While remembering the event, I feel as though I am mentally reliving it : 1 = not at all, 7 completely
	*Location*	I remember the location where the event took place: 1 = not at all clear, 7 = very clearly
	*Time*	I remember the time of the day whern the event took place: 1 = not at all clear, 7 = very clearly
	*Coherence*	While remembering the event, it comes to me as a coherent story and not as an isolated scene: 1 = not at all, 7 = completely.
***Self-referential Information (SRI)***	*One's own actions*	I remember what I did during this event: 1 = not at all, 7 = very clearly.
	*One's own words*	I remember what I said during this event: 1 = not at all, 7 = very clearly.
	*One's own thoughts*	I remember what I thought during this event: 1 = not at all, 7 = very clearly.
	*Visual perspective*	Previous studies have shown that people can report that they can visualize different memories from different points of view. Using the bellow mentionned categories, from which point of view do you see your self? A) In your memory, you imagine the scene as an observer could see it. As an observer, you can see yourself and other aspects of the situation. B) In your memory, you imagined the scene from your own point of view (through you own eyes). You are an actor. C) Any of the above mentionned perspectives described the way you remember the situation. At which point are you observer or actor in the situation: 1 = totally observer; 7 = totally actor
***Emotionality***	*Valence*	When the event happened, my emotions were: 0 = very negative, 7 = very positive.
	*Personal importance*	This event is important to me (it involves an important theme or episode in my life): 1 = not at all important, 7 = very important.
	*Feeling emotions*	While remembering the event, I feel the emotions I felt when the event occurred: 1 = not at all,7 = completely.
***Reactivation***	*Reactivation frequency*	Since it occurred, I have thought or talked about this event: 1 = not at all, 7 = very often.
***Confidence***	*Real/imagine*	I believe the event in my memory really occurred in the way I remember it and that I have not imagined or fabricated anything that did not occur: 1 = 100% imaginary, 7 = 100% real.

### Analysis

Paired sample Wilcoxon test was used in order to determine if target memory of each 4 groups contains the same quantity of characteristics than other memories. Non-parametric Kruskall-Wallis test was used to assess the group effect for each of the 5 types of memories (recent and old real, recent and old imagined, and target memories). Mann Whitney U test was used as post-hoc test following Kruskall-Wallis analyses that revealed a significant difference. Comparisons were first made on the MCQ total scores and then on each of the 6 categories subscores (i.e., sensory details, memory clarity, self-referential and emotional information, reactivation frequency, confidence in their own memory).The alpha level was set at 0.05. Data were analyzed using SPSS 16.0 (2007, SPSS Inc.).
